# A 4-Decade Population-Based Registry of Thoracic Aortic Dissection Causing Sudden Death in the Young

**DOI:** 10.1016/j.jacadv.2025.102487

**Published:** 2025-12-30

**Authors:** Monica De Gaspari, Cristina Basso, Francesca Dalla Zanna, Maria Bueno Marinas, Marco Cason, Rudy Celeghin, Serena Pinci, Martina Perazzolo Marra, Domenico Corrado, Ugo Fedeli, Hector I. Michelena, Stefano Nistri, Gaetano Thiene, Kalliopi Pilichou, Stefania Rizzo

**Affiliations:** a Cardiovascular Pathology Unit, Azienda Ospedale-Università Padova, Italy; b Department of Cardiac, Thoracic, Vascular Sciences and Public Health, University of Padua, Padova, Italy; c Epidemiological Department, Azienda Zero, Veneto Region, Padova, Italy; d Department of Cardiovascular Medicine, Mayo Clinic, Rochester, Minnesota, USA

**Keywords:** aortic dissection, bicuspid valve, connective tissue disease, genetic, histopathology

## Abstract

**Background:**

Thoracic aortic dissection (TAD) causing sudden cardiac death (SCD) in the young is overlooked and poorly described.

**Objectives:**

The purpose of this study was to assess: 1) the incidence of TAD-related SCD, and 2) the prevalence and features of TAD in a young cohort with SCD.

**Methods:**

The number of residents ≤40 years in the Veneto Region, Italy (1985-2024) was calculated, and the SCD registry was searched for TAD. Clinical features and multilevel aortic diameters were assessed. Aortic histopathology was performed in all and whole exome sequencing when feasible.

**Results:**

The overall rate of TAD-related SCD in this young cohort (age 1-40 years) was 0.32 per 1,000,000 (95% CI: 0.21-0.46) people. Twenty-eight out of 941 SCD (2.9%; mean age 29.1 ± 7 years; 24 males) had TAD, including 24 type A and 4 type B. 7 (29%) also had a chronic TAD. Symptoms were present in 13 (46%) subjects. Associated conditions were bicuspid aortic valve (BAV 9, 6 with early-complex pattern including aortic coarctation in 2), hypertension (5), Marfan syndrome (2), pregnancy (2), isolated aortic coarctation (2), and Turner syndrome, previous closure of ductus arteriosus, familial TAD, and drug abuse (1 each). Genetic abnormalities were found in 8/16 TAD (50%), including 1/3 screened BAV. In type A TAD, aortic diameters were<50 mm in all but 2 cases. A heterogeneous histopathological pattern was found.

**Conclusions:**

TAD-related SCD in the young is rare, and TAD is an infrequent cause of SCD, affecting mostly men. Symptoms and chronic TAD are common, and a high index of clinical suspicion is needed for timely diagnosis. BAV (primarily the complex-phenotype) and inherited disorders are the most common associated conditions, supporting the role of routine genetic screening in this population.

Dissection of the thoracic aorta (TAD) is the most lethal condition involving the aorta, which occurs mostly in males and in the seventh decade of life.^1^ After clinical presentation, survival is time-dependent so that prompt diagnosis is crucial for a favorable prognosis.^1^, ^2^, ^3^, ^4^ However, not all TAD cases present alive since some present with sudden cardiac death (SCD) as the first manifestation of the underlying aortopathy.

Studies of SCD in the young (ie, <35-40-year-old patients) focused their attention on coronary artery disease and cardiomyopathies,^5^, ^6^, ^7^, ^8^ while TAD-related SCD was neglected with the exception of a couple of studies.^9^^,^^10^ However, only a Spanish group reported Marfan and Marfan-like syndromes, cocaine use, coarctation of the aorta, and bicuspid aortic valve (BAV) as clinical correlate.^9^ BAV is the most frequent congenital cardiac anomaly^11^, ^12^, ^13^ with a high morbidity burden during the lifetime, mostly due to valvulopathy and aortic aneurysm requiring surgery, as shown by the largest population-based study of echocardiography-diagnosed BAV with the longest follow-up to date. Importantly, TAD was overall infrequent in that cohort (1.6% by age 90) and absent before the age of 40.^14^

In the Veneto Region, Northeast Italy, we have been prospectively accruing cases of SCD in the young for approximately 4 decades, thus having the unique opportunity to evaluate the burden and clinicopathological correlates of TAD in this specific population. This is of relevance, since TAD is an uncommon cause of SCD in this age segment and the issue of associated conditions is unresolved. Thus, we aimed at assessing: 1) the incidence of TAD-related SCD within a geographically defined population as well as the prevalence of TAD in a young cohort with SCD as identified at autopsy; 2) the clinical condition before death; 3) the associated risk factors for TAD; and 4) the histopathological analysis of the aortic tunica media and its relationship to associated conditions and aortic size.

Precise data on prevalence and clinical presentation of TAD in young SCD victims could impact clinical practice and policymakers. The existence of prodromic symptoms or signs could raise the index of clinical suspicion for timely diagnosis. The presence of associated risk factors could help early identification and prevention of TAD and SCD in apparently healthy young people.

## Material and methods

To assess the incidence of TAD-related SCD within a geographically defined population, we used a prospective young (≤40 years old) population-based SCD registry from the Veneto Region and estimated the incidence TAD-related SCD. We also calculated the prevalence of TAD among the cases of SCD as identified at autopsy. For this purpose, the denominator was the number of residents 1 to 40 years old in the Veneto Region, Northeast Italy, January 1985-December 2024, obtained from the Regional Epidemiological Center, and the numerator was collected from the 4-decade-long SCD registry. STROBE (Strengthening the Reporting of Observational Studies in Epidemiology) guidelines were used to ensure the reporting of this observational study.^15^

### Population

The Cardiovascular Pathology Unit, University Hospital, Padua, has been the single-unique referral center of the Veneto Region for the study of SCD in the young (patients< 40 years) since 1985. The Veneto Region is a well-defined geographic area of 18.399 km^2^ with a population of approximately 4.8 million inhabitants. According to the Regional Health System, every case of SCD occurring in young people should undergo autopsy, and the heart/aorta is referred to our unit which works as Core Lab. All consecutive autopsy cases of SCD in young patients were studied according to an established protocol so that the cardiovascular specimen was sent to the Padua Pathology Core Lab, after the exclusion of extracardiac causes.^16^ The autopsy rate of SCD in the young is estimated above 95% thus being representative of the actual target population. SCD refers both to out-of-hospital deaths and to patients transported to the hospital who subsequently died. Patients who initially experienced ventricular fibrillation and were revived by cardiopulmonary resuscitation or by a single automated external defibrillator (AED) shock were included in our definition of SCD. If the patients survived transiently but subsequently died in hospital, they were still classified as SCD and subjected to autopsy.

This 4-decade Juvenile SCD Registry was thus searched for TAD cases. TAD was classified as type A (involves the ascending aorta and/or aortic arch, and possibly the descending aorta; the tear can originate in the ascending aorta, the aortic arch, or more rarely, in the descending aorta) and type B (involves the descending aorta or the arch, distal to the left subclavian artery, without the involvement of the ascending aorta).^17^ The final cause of death was described considering all possible complications such as external rupture with hemopericardium or hemothorax or hemoretroperitoneum, aortic incompetence, coronary artery involvement, organ ischemia, stroke, etc. We collected information on medical history, circumstances of death, external examination, complete autopsy, and toxicological analysis. The study complied with the Declaration of Helsinki. The gross and histologic samples were used in accordance with the “Recommendation (CM/Rec [2016] 6) of the Committee of Ministers to Member States on research on biological materials of human origin,” released by the Council of Europe, as received by the Italian National Council of Bioethics. The registry is approved by the Regional Review Board, and no ethical issues are involved. The data that support the findings of this study are available from the corresponding author on reasonable request.

### Gross description of the heart and aorta

All cardiovascular specimens of juvenile SCD are stored in the Registry of the Cardiovascular Pathology Unit. In each cardiovascular specimen, the presence of aortic coarctation and BAV was assessed. The intimal tear was described in terms of disposition (horizontal vs longitudinal) and distance from the aortic valve commissure. The type of BAV was defined according to the 2021 International Consensus Classification and Nomenclature for the congenital BAV condition.^18^

Ascending aorta internal circumferences were measured with a flexible ruler at 4 levels, that is, the annulus, the sinuses of Valsalva, the sinotubular junction, and the proximal ascending aorta. The measurements were made perpendicular to the long axis of the aorta, as previously reported in different echocardiographic studies which investigated aortic root dilatation.^12^^,^^19^ The aortic diameters were calculated with the formula: *diameter = circumference/π*. To correct for the possible relative underestimation of the “in vivo” aortic size due to the absence of perfusion in the aortic specimens, a coefficient (lq = 1.2) was then applied to all the aortic measurements, as previously reported.^20^

Chronic aortic dissection is characterized by a false lumen which is often partially or completely thrombosed and endothelialized, giving it a smooth, glistening lining that mimics a true lumen, and by a usually thickened, stiff, and fibrotic intimal flap.

### Histopathological analysis

The study protocol of cardiovascular specimens includes routine sampling of the aorta, and for each case formalin-fixed paraffin-embedded (FFPE) tissue is available in the archives of the Cardiovascular Pathology Unit. Multiple samples of the proximal ascending aorta were obtained in each case, fixed in 10% buffered formalin (pH 7.35) and embedded in paraffin. Five microns thick, FFPE sections were serially cut and stained by hematoxylin–eosin and Heidenhain trichrome. Weigert-van Gieson, Alcian–periodic acid–Schiff, and assessed under light microscopy (Carl Zeiss, Zeiss Plan-Neofluor lenses) and digitally acquired. The aortic tunica media was independently analyzed by 2 observers (S.R. and M.D.G.), blinded to the patient's information. The following parameters were assessed: elastic fiber fragmentation (EFF) and/or loss, smooth muscle cell nuclei loss (SMCNL), mucoid extracellular matrix accumulation (MEMA), either intralamellar or translamellar.^21^ The severity and extent of medial degenerative changes were scored on a scale of 0 to 3 (0 = none, 1 = mild, 2 = moderate, and 3 = severe and 0 = none, 1 = focal, 2 = multifocal, and 3 = extensive), except for SMCNL severity, which was scored on a scale of 0 to 2 (0 = none, 1 = patchy, and 2 = bands), and of MEMA, which was categorized into 2 types (intralamellar and translamellar), and was scored on a scale of 0 to 6 for both extent and severity by multiplying the extent and severity by 1 for cases with intralamellar MEMA and by 2 for cases with translamellar.^22^ Scoring disagreements were adjudicated by the senior cardiovascular pathologist (C.B.).

Chronic aortic dissection is defined histologically by a two-layered, atrophic tunica media with fibrous thickening of the false lumen and by an usually hyperplastic neointima.

### Genetic testing

Genomic DNA isolation was performed from three 10-μm FFPE sections of the aorta and/or myocardium with the use of QIAamp DNA FFPE Tissue Kit (Qiagen) according to the manufacturer’s protocol. DNA concentration was measured with Qubit Broad Range Kit (Thermo Fisher Scientific). Whole exome sequencing was performed on Illumina NextSeq platform as previously reported.^23^

Gene-disease selection related to hereditary thoracic aortic aneurysm and dissection was based on the results of Clinical Genome Resource (ClinGen) framework.^24^

Variants were described based on the current Human Genome Variation Society genetic variant nomenclature guidelines using the accession number of the longest transcript, and the interpretation and classification of these variants were performed according to the current American College of Medical Genetics and Genomics recommendations.^25^

### Statistical analysis

Continuous variables are expressed as either mean ± SD or median with a 95% (CI as appropriate. Kruskal-Wallis test was used to compare data from the following groups: BAV, Marfan, hypertension, pregnancy, and others. We also performed Kruskal-Wallis test followed by Dunn’s post hoc multiple comparisons, comparing the mean rank of each group with the mean rank of every other group. A value of *P* < 0.05 was considered statistically significant.

Death rates (per 1,000,000) through the whole 1985 to 2024 study period were computed with the Veneto Region population aged 1 to 40 years retrieved from the Italian National Institute of Statistics website (https://demo.istat.it/). Ninety-five percent CIs were estimated based on the Poisson distribution of observed deaths.

## Results

### Study population

In the time interval 1985-2024, the overall rate of SCD was 10.8 per 1,000,000 (95% CI: 10.1-11.5) inhabitants, 1 to 40 years old, in the Veneto Region, Italy. The overall rate of TAD-related SCD was 0.32 per 1,000,000 (95% CI: 0.21-0.46). Among the total population of 941 SCD spanning 40 years of population-based SCD referrals, 28 had TAD (prevalence of 2.9%), mean age was 29.1 ± 7 years, 24 males, and 4 females. 7 cases (25%) were referred from forensic autopsies and 21 from clinical autopsies. Of them, 24 had type A TAD and 4 type B TAD (Table 1). Circumstances of death were at rest in 24 (86%) and on exercise in 4 (14%). Two were athletes who had previously undergone annual preparticipation screening for sport activity with normal results.

**Table 1:** TAD-Related SCD Study Population: Main Demographic, Clinical, Autopsy, and Genetic Data

#	Age, y	Sex	Previous Symptom, days, ER	In Vivo Diagnosis	Circumstances Sport Activity	TAD	TAD Complications	Chronic TAD	Associated Condition	BAV Type, R, Symm/Asymm, nr Sinus	An	Sin	Sin-tub	Tub	Genetic Test	Gene	Mutation/Chrom abn
1	21	M	CP, 7	0	Rest	A	Hemopericardium, aortic incompetence	0	BAV	Fused, R, Symm, 3	27.9	30.6	28.3	34.4	NA		
2	24	M	CP, 3	0	Effort	A	Hemopericardium	0	BAV	Fused, R, Asymm, 3	30.7	35.2	34.4	32.2	NA		
3	33	M	Aortic murmur, >2,000	1 (BAV, 44 mm Hg, 38 mm sinusal)	Rest	A	Hemopericardium	0	BAV (complex)	Fused, R, Asymm, 3	32.5	39.2	36.3	34.4	NA		
4	35	M	-	0	Rest	A	Hemopericardium	1	BAV (complex)	Fused, R Symm, 3	34.4	43.2	42.2	55.4	0		
5	27	M	CP, 2, ER	0	Sleep	A	Hemopericardium	1	BAV	Fused, R Asymm, 3	32.6	38.2	36.3	40.2	NA		
6	36	M	CP, 6, ER	0	Rest	A	Hemopericardium	0	BAV (complex)	Fused, R, Asymm, 3	34.4	48.2	45.8	45.2	1	FBN1	c.6005C>T
7	24	M	CP, 1	0	Sleep	A	Hemopericardium	1	BAV (complex)	Fused, R, Asymm, 3	31.3	47.8	45.6	48.2	0		
8	22	M	HTN, >700	1 (CoA, surgery, 37 mm sinusal)	Rest	A	Hemopericardium	0	BAV+ CoA (complex)	Fused, R Asymm, 3	30.6	36.2	34.4	45.6	NA		
9	17	M	Hematemesis, 1	0	Rest	A	Hemopericardium	0	BAV+ CoA (complex)	Fused, R Asymm, 3	29.8	34.4	33.2	43.2	NA		
10	28	M	CP, faintness, vomit, 1 ER	1 (Marfan)	Rest Athlete (basketball)	A	Hemopericardium, coronary ostia involvement, aortic incompetence	0	Marfan	-	32.5	42.2	42.2	45.4	1	FBN1	c.4011delT
11	31	M	-	1 (Marfan)	Rest	A	Hemopericardium	0	Marfan	-	36.3	50.2	49.2	51.2	1	FBN1	c.5930G>A
12	29	M	CP, 10	0	Rest	A	Hemopericardium	1	Family history	-	25.2	29.0	24.8	48.2	1	NOTCH1	c.4401C>A
13	27	F	-	0	Rest	A	Hemopericardium	0	Pregnancy	-	26.5	33.4	30.2	28.2	NA		
14	30	F	Dyspnea, syncope, 3 ER	0	Rest	A	Hemopericardium	1	Pregnancy	-	26.3	34.2	31.2	32.4	1	COL3A1	c.3005G>A
15	35	M	CP, 1	0	Rest	A	Hemopericardium	0	HTN	-	26.4	36.3	35.4	31.3	NA		
16	32	M	-	0	Rest	A	Hemopericardium	0	HTN	-	32.4	40.5	38.9	27.5	0		
17	40	F	-	0	Rest	A	Hemopericardium	0	HTN	-	27.2	28.6	27.3	27.8	0		
18	39	M	CP, 1	0	Rest	A	Hemopericardium	0	HTN	-	32.4	34.7	34.7	35.0	0		
19	40	M	-	1 (CKD, HTN)	Rest	A	Hemopericardium	0	HTN	-	35.9	42.8	39.0	40.0	0		
20	33	M	-	0	Rest	A	Hemopericardium	0	Drug addict	-	35.1	37.4	36.7	34.8	0		
21	21	M	-	0	Rest	A	Hemopericardium	0	PDA closure	-	23.0	29.4	25.9	35.1	NA		
22	17	M	-	0	Rest Athlete (orienteering)	A	Hemopericardium	1	None	-	24.4	28.5	26.3	38.9	1	TGFBR1	c.1472G>A
23	38	M	-	0	Rest	A	Hemopericardium	0	None	-	27.5	32.4	29.8	30.2	0		
24	39	M	-	0	Effort	A	Hemopericardium	1	None	-	24.5	32.7	42.2	47.1	1	MYLK	c.4415+1G>C
25	27	M	-	1 (CoA, surgery)	Rest	B	Hemothorax	0	CoA	-	NA	NA	NA	NA	NA		
26	22	M	-	0	Effort	B	Hemothorax	0	CoA	-	NA	NA	NA	NA	NA		
27	13	M	-	0	Effort	B	Hemothorax	0	None	-	NA	NA	NA	NA	NA		
28	34	F	-	1 (Turner)	Rest	B	Hemothorax	0	Turner	-	NA	NA	NA	NA	1		45X0

An = annulus; Asymm = asymmetric; BAV = bicuspid aortic valve; Chrom abn = chromosome abnormalities; CKD = chronic kidney disease; CoA = isthmic coarctation; COL3A1 = collagen type III alpha 1 chain; CP = chest pain; ER = emergency room; FBN = fibrillin; HTN = hypertension; MYLK = myosin light chain kinase; NOTCH1 = notch receptor 1; Nr = number; PDA = patent ductus arteriosus; R = raphe, sin = sinusal; Symm = symmetric; tub = tubular; TGFBR1 = transforming growth factor beta receptor 1; TAD = thoracic aortic dissection.

Symptoms in the week preceding SCD were reported in 13 (46%), consisting of precordial and/or back pain in 8. Four of them had an emergency room access, one to 7 days before the fatal event: based on negative clinical examination, laboratory tests, chest x-ray, and 12-lead electrocardiogram (ECG), the patients were discharged with a diagnosis of thoracodynia.

Conditions associated with type A TAD included BAV in 9 (37.5%) patients of whom 6 had complex BAV (associated with aortic coarctation in 2 including one with previous aortic surgery, accelerated valvulo-aortopathy in 3 patients, and associated fibrillin gene genetic abnormality in 1 patient) while the remaining 3 had the typical uncomplicated BAV; previously diagnosed arterial hypertension in 5; previously diagnosed Marfan syndrome in 2 (both under beta-blockers therapy, though collected in the late 80s, thus out of the current follow-up management strategies); pregnancy (third trimester) in 2; previous closure of patent ductus arteriosus, family history of TAD or of cocaine abuse one each; and 3 type A TAD were classified as idiopathic. Type B TAD was associated with aortic coarctation in 2 (one diagnosed during life with previous surgery), known Turner syndrome in one, while no associated condition was detectable in one patient.

Overall, an in vivo diagnosis of the associated condition was available in 7 (25%) patients: 2 BAV (one with previously operated aortic coarctation), 2 Marfan syndrome, 1 Turner syndrome, 1 with previously operated patent ductus arteriosus, and 1 with previously operated aortic coarctation.

### Type A TAD: macroscopic examination

The intimal tear was localized in the ascending aorta in all type A TAD, mostly horizontally oriented (16 cases, 67%), with semicircumferential or circumferential extension, and at a distance of 0 to 20 mm (mean 0.67 ± 0.65 mm) from the aortic valve commissure (ie, sinotubular junction). 7 cases (29%; age range 27-39 years; mean 28.7 ± 7; 6 females) showed also a previously unrecognized chronic TAD.

**Figure 1:**
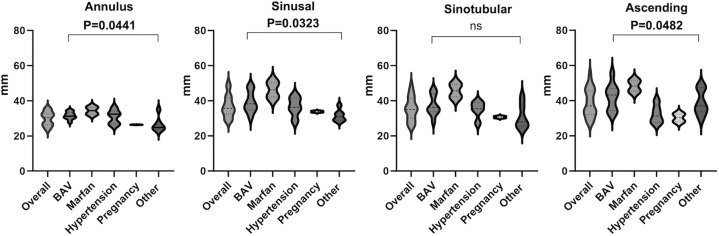
**Aortic Diameters at the 4 Levels** The measurements are represented in the entire TAD population and in the 5 subgroups. Violin plots are used to visualize the distribution (median and quartiles) at 4 levels (annulus diameter *P* = 0.0441; sinusal diameter *P* = 0.0323; sinotubular junction diameter P = NS; ascending diameter *P* = 0.0482). Values are expressed as median ± 95% CI. BAV = bicuspid aortic valve; TAD = thoracic aortic dissection.

**Figure 2:**
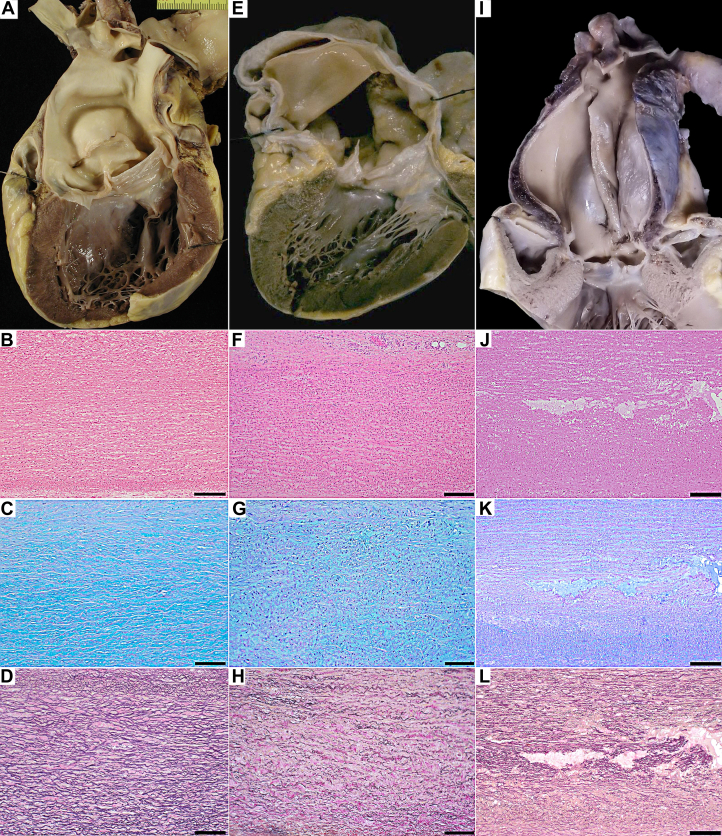
**SCD in Young Patients With Type A TAD** (A) Asymptomatic 36-year old young adult with type A acute TAD, BAV, and positive postmortem genetic (FBN1 -c.6005C>T; p.(Pro2002Leu) (case#6). (B-D) Mild degenerative changes of the tunica media (hematoxylin-eosin bar 200 m, Alcian PAS bar 100 m, and Weigert van Gieson stain bar 100 m). (E) Asymptomatic 35-year-old young adult with type A acute and chronic TAD, BAV, and negative postmortem genetic screening (case#4). (F-H) Mild-moderate degenerative changes of the tunica media (hematoxylin-eosin bar 200 m, Alcian PAS bar 100 m, and Weigert van Gieson stain bar 100 m); (I) Asymptomatic 17-year-old boy with type A acute and chronic TAD without known risk factors and positive postmortem genetic screening (TGFBR1 -c.1472G>A; p.(Arg491Gln) (case#22). (J-L) Severe degenerative changes of the tunica media with predominant elastic fibers fragmentation (hematoxylin-eosin bar 200 m, Alcian PAS bar 200 m, and Weigert van Gieson stain bar 200 m). FBN = fibrillin; SCD = sudden cardiac death; other abbreviations as in Figure 1.

The aortic diameters measured in the entire TAD population (overall) and for each associated condition are reported in Figure 1 as median ± 95% CI. A wide variability among groups was present that reached the statistical significance. Marfan syndrome-TAD had large aortic diameters at the sinusal, sinotubular, and tubular level, and one of them reached 50.2 and 51.2 mm at the sinusal and tubular level, respectively. Only one BAV-TAD had a tubular aorta diameter of 55 mm (likely accelerated aortopathy), in the setting of previously unrecognized chronic dissection (Figure 1). Cases of type A TAD-SCD are represented in Figure 2.

In the 9 patients with BAV-TAD, the BAV showed an anteroposterior disposition with a raphe in the anterior cusp in all, that is, fused BAV with right-left cusp fusion and raphe. Calcification was absent in all. Mild thickening of the free edge of the cusps was present in 3. BAV had been diagnosed in 2 patients at the age of 20 and 26 years (age at SCD 22 and 33 years, respectively), and the last echocardiograms had been performed, respectively, 18 and 34 months before SCD, documenting mild aortic incompetence in both, and maximum ascending aorta diameter of 37 mm and 38 mm, respectively. At postmortem, the maximum aortic diameter was 39 mm (sinus level) and 45 mm (tubular level), respectively. Cusp fibrous thickening was visible only in the case diagnosed in vivo as moderate-to-severe aortic stenosis.

### Type A TAD: tunica media histopathological evaluation

**Figure 3:**
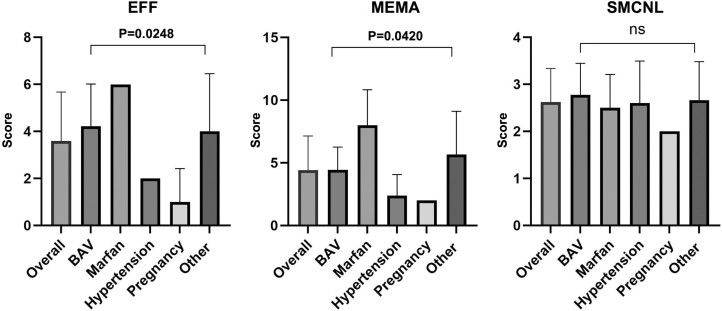
**Histopathologic Changes of the Aortic Tunica Media** Values of EFF, SMCNL, and MEMA are represented in the entire TAD population and in the 5 subgroups (EFF *P* = 0.0248; SMCNL P = NS; MEMA *P* = 0. 0420). Values are expressed as mean ± SD. EFF = elastic fiber fragmentation; MEMA = mucoid extracellular matrix accumulation; SMCNL = loss of smooth muscle cell nuclei; other abbreviation as in Figure 1.

The EFF, SMCNL, and MEMA in the different groups are shown in Figure 3 as mean ± SD. Atherosclerosis was reported only in one case (hypertensive TAD). Inflammation was not identified. In the 2 Marfan-TAD patients, the EFF was severe and the MEMA appeared typically in the form of lacunae or wide buildup of mucopolysaccharides (translamellar MEMA). SMCNL was patchy rare in one and frequent in the other. Otherwise, in the 5 hypertensive TAD cases, EFF was always mild, MEMA usually focal and intralamellar and the prevalent changes consisted of SMCNL, always patchy, rare in 3 cases, and frequent in 2. In the 9 BAV-TAD patients, the EFF pattern was the most prevalent histopathologic degenerative change (absent in only 1) and was heterogeneous, being mild in 2, moderate in 3, and severe in 4. MEMA could be both intralamellar and translamellar but never severe. SMCNL was frequent (6 cases) and patchy in all but one with a band-like appearance.

Overall, a wide variability among groups was found that reached the statistical significance in terms of EFF and MEMA.

### Genetic screening

Retrospective genetic screening on available FFPE tissue sections or in vivo genetic screening was feasible in 16 out of 28 TAD cases (57%): 4 with hypertension, 3 with BAV, 3 idiopathic, 2 with Marfan syndrome, and one each with familial TAD, Turner syndrome, pregnancy, and drug abuse, respectively. According to the methods (https://search.clinicalgenome.org/kb/affiliate/10044?page=1&size=25&search=), pathogenic/likely pathogenic variants in BAV or connective tissue-related genes were identified in seven (Supplemental Table), plus a full X monosomy in the patient with Turner syndrome accounting for 8/16, 50% positive genetic test. The 2 cases with an in vivo diagnosis of Marfan syndrome were, respectively, carrying: 1) the fibrillin-1 gene missense variant c.5930G>A, which is causing the aminoacidic substitution p.(Cys1977Tyr) in the epidermal growth factor 34 domain of the fibrillin protein; and 2) the fibrillin-1 gene single-nucleotide deletion c.4011delT, which is causing a frameshift p.(Val1338TyrfsTer75), with subsequent premature truncation at the epidermal growth factor-like 22 domain of the fibrillin protein, respectively.

Another fibrillin-1 gene missense variant, c.6005C>T was identified in one patient with BAV, whereas the other 2 BAV cases investigated remained gene elusive. This variant is causing the aminoacidic substitution p.(Pro2002Leu) in the epidermal growth factor 34 domain of the protein. In another patient with a family history of TAD and tricuspid aortic valve, the rare *NOTCH1* variant c.4401C>A was identified, leading to a premature termination of the protein, p.(Cys1467Ter), at the Lin-12/Notch repeat 1 domain. Similarly, a α-1 procollagen type III gene heterozygous missense variant, c.3005G>A was found in a pregnant woman with TAD, leading to a single aminoacid substitution, p.(Gly1002Asp), in the triple-helical region. In the 2 patients with the so-called “idiopathic” TAD, the missense transforming growth factor beta receptor type I gene variant c.1472G>A, changing the arginine residue to glutamine (p.[Arg491Gln]); and the myosin light chain kinase gene splicing site variant c.4415+1G>C leading to premature termination of the protein, were identified.

Finally, none of the 4 screened cases with hypertensive TAD had a positive genetic test.

## Discussion

### TAD as a cause of SCD in young patients

Our findings demonstrate that TAD-related SCD is quite a rare event in the young (age 1-40 years) with an incidence of 0.32 per 1,000,000 inhabitants. The occurrence of TAD was often preceded by symptoms and, surprisingly, accompanied by a chronic dissection in one-third of patients with type A TAD. The fact that 14% of our patients had an emergency room access, one to 7 days before the fatal event, and were discharged with a diagnosis of thoracodynia based on negative clinical, laboratory, ECG, and imaging testing, is consistent with other studies. Morentin Campillo et al^9^ found that in one-third of TAD-SCD cases was preceded by symptoms of chest/back pain. This finding highlights the need for an increased awareness of TAD as a cause of chest pain in young individuals,^26^ which is however challenged by its rare occurrence in this age group.

The 2.9% prevalence of TAD among SCD in people aged <40 years is aligned with other studies on SCD in the young, with rates varying from 1.9% to 6.7%.^5^, ^6^, ^7^, ^8^, ^9^, ^10^ However, the incidence of TAD-SCD in the young population and clinical features has not been well or sufficiently studied in geographically defined areas or population-based studies. In most previous studies,^5^, ^6^, ^7^, ^8^^,^^10^ there is no mention of the associated conditions to TAD, with the possible exception of Marfan and Marfan-like syndromes. A Spanish study^9^ found that the most common associated condition was a BAV, occurring in 43% of their population. In our study, we also found BAV to be the most frequent one, occurring in 32% of patients.

Our series demonstrates that inherited connective tissue disorders and a fused-type BAV, with right-left cusp fusion and raphe (combined with either a significant valvular heart disease or aortic coarctation in only 2 cases), are the most common conditions associated with type A-TAD in SCD, besides arterial hypertension. In the International Registry of Aortic Dissection (IRAD), Januzzi et al^4^ showed that young patients with TAD have unique risk factors when compared with older patients. In particular, Marfan syndrome and BAV were often observed, the latter representing 9% of all cases in their subgroups of 68 patients aged <40 years, yet Marfan being more common. In our Registry of juvenile SCD, we confirm and further expand these data, with BAV being identified in more than one-third of young people who died suddenly with type A TAD, without known syndromic features (Central Illustration).

**Central Illustration:**
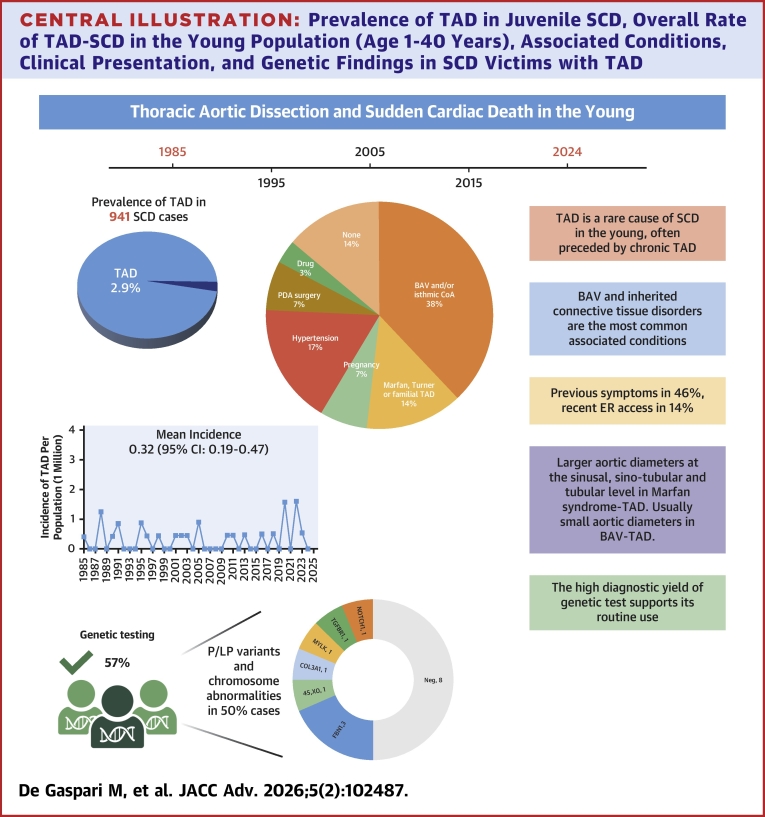
**Prevalence of TAD in Juvenile SCD, Overall Rate of TAD-SCD in the Young Population (Age 1-40 Years), Associated Conditions, Clinical Presentation, and Genetic Findings in SCD Victims with TAD** CoA = isthmic coarctation; COL3A1 = collagen type III alpha 1 chain; ER = emergency room; FBN = fibrillin; MYLK = myosin light chain kinase; NOTCH1 = notch receptor 1; PDA = patent ductus arteriosus; TGFBR1 = transforming growth factor beta receptor 1; other abbreviations as in Figures 1 and 2.

While TAD is even more rare in women (only 4 cases), 50% of them were pregnant. This is consistent with the knowledge that pregnancy increases the risk of aortic events across childbearing women with or without various predisposing factors (eg, hypertensive disorders of pregnancy) as recently shown by Chen et al^27^ who could determine pregnancy to be an independent risk factor.

### The growing importance of genetics in young patients with TAD-SCD

Syndromic conditions were present in 3 patients, that is, one with Turner syndrome and 2 with Marfan syndrome. Pathogenetic variants or chromosomal abnormalities could be detected in 8 out of the 16 patients in whom genetic testing could have been performed. Though we cannot rule out the presence of syndromic features in these individuals with certainty, it is noteworthy that our diagnostic yield (50%) is higher than that generally reported, that is, 25%.^24^ This finding highlights the importance of the genetic testing in individuals experiencing TAD, particularly if young. In fact, it has been reported that the same genes responsible for syndromic TAD may also have highly penetrant TAD variants that present without other syndromic features,^24^ consistently with the present findings. At least 11 genes have been confirmed to be associated with thoracic aortic aneurysms and TAD, with or without syndromic forms. These genes encode proteins that are involved in vascular SMC contraction and adhesion to the extracellular matrix, transforming growth factor beta signaling pathway, or SMC metabolism. Pathogenic/likely pathogenic variants in these genes can be identified in the majority of TAD families with systemic features of Marfan or Loeys-Dietz syndromes, but in the absence of syndromic features, the detection rate of gene variants is reduced to 30% of TAD families, suggesting that other still unknown genes need to be identified.^28^

Recently, molecular testing of familial TAD-associated genes has been shown to be a highly effective tool to identify the genetic cause of TAD-SCD and benefits surviving at-risk families.^29^ Our data are in keeping with the literature, suggesting a high diagnostic yield of genetic screening in young victims of TAD, with pathogenic/likely pathogenic variants or chromosomal abnormalities in half of tested cases, thus supporting the recommendation for postmortem genetic test.^30^ In our limited cohort, only hypertensive TAD patients remained gene elusive. Most of TAD cases carried pathogenic variants in extracellular matrix and transforming growth factor pathway genes, that is fibrillin1, myosin light chain kinase, collagen type III alpha 1 chain, and transforming growth factor beta receptor 1 genes. Moreover, the identification of notch receptor 1 gene pathogenic variant in a TAD patient with tricuspid aortic valve confirms that the alteration of this gene is a novel cause of thoracic aortic aneurysm alone and not as a result of the BAV, as recently reported in the literature.^31^ Noteworthy, 2 out of the 3 TAD cases without “risk factors” and who would have been called “idiopathic” revealed to carry a pathogenic/likely pathogenic variant of myosin light chain kinase and transforming growth factor beta receptor 1 genes, respectively.

### The relationship between BAV and TAD

The link between BAV and TAD has been put forward on the basis of historical studies overestimating the prevalence of BAV in TAD, reaching up to 28%.^32^, ^33^, ^34^, ^35^ This relationship led to suggest early surgical therapy in BAV individuals, recommendation which has been subsequently modified. Indeed, the prognosis of asymptomatic patients with BAV has been demonstrated to be good with observed survival similar to the expected one.^36^^,^^37^ In a recent large community-based cohort of BAV, with a long-term follow-up, TAD was reported in 6 out of 652 individuals, all males, aged 47 to 75 years and in one 75-year-old male out of 12 patients who died resulting in a lifetime cumulative TAD incidence of 1.6%.^14^

Our observation that BAV is found in 37.5% of type A TAD-SCD and 32% of all TAD-SCD at a young age should not be misinterpreted and TAD should not be attributed to BAV per se. The present data provide further evidence that it is BAV patients with complex-phenotypes who deserve particular attention.^18^ Our findings should indeed be interpreted within this conceptual framework since 6 out 9 BAV patients in our study fully satisfy criteria of the early-onset complication pattern. Importantly, in the largest and longest natural history study available to date in BAVs, TAD was not reported even in young individuals with complex-presentation. Moreover, the role of rare copy number variants in nonsyndromic BAV, particularly in cases with early onset or complex presentations, has been demonstrated. As a novel contribution, our findings confirm and expand historical findings suggesting that a minority of early-complicated BAV patients are prone to a rare, but ominous complication such as TAD. These findings pose a novel, challenging piece in the puzzle of BAV aortopathy for the vast majority behaving more like the general population than hereditary connective tissue disorders.^2^^,^^14^^,^^38^ Noteworthy, the Spanish study also found that there are some uncomplicated BAV cases presenting with SCD-TAD^9^ and in the original study by Edwards et al^32^ 5 of 9 BAV-TAD had apparently uncomplicated typical BAV, although 50% of BAV-TAD had associated mitral valve prolapse suggestive of a not specific connective tissue disease. Overall, genetic data were not available in these previous studies and, since genetic testing was feasible only in 3 out of 9 BAV-TAD cases in our series, we cannot exclude that more genetic disease could have missed in these BAV-TAD, so that the so-called “uncomplicated BAV” could be overrepresented.

### Aortic diameters and risk of TAD

The risk for TAD is routinely assessed with echocardiographic follow-up studies by measuring the aortic diameter, according to the most recent guidelines.^2^^,^^3^ In our SCD-TAD series, aortic size measured at postmortem were well below 50 mm in 98% of patients. In the International Registry of Aortic Dissection, by excluding those with Marfan syndrome, TAD occurred with a mean aortic diameter below 50 mm at all level except for the ascending aorta (mean value 53 + 19 mm),^1^ establishing the conceptual framework of the aortic paradox, that is, many dissections occur below the prophylactic surgical threshold for intervention.^39^

Clinical outcomes for BAVs have been shown to be consistently better than in patients with Marfan syndrome.^40^ In a large population from the International Bicuspid Aortic Valve Consortium of BAV patients with ascending aorta 50 to 54 mm under surveillance, TAD incidence was low, and the overall rates of TAD and surgical mortality were similar, suggesting clinical equivalence between surgical and surveillance strategies.^41^ Guidelines both from United States and Europe recommend the same aortic surgery threshold independent of aortic valve morphology.^2^^,^^3^

Although the small sample size of Marfan patients does not allow to obtain statistically significant values, in our series BAV-TAD had also lower aortic diameters at all levels. Noteworthy, the location of maximal aortic dilation, always within the ranges of no surgical indication, occurred at the tubular portion in BAV, with or without sinusal involvement, at difference from Marfan with the larger values at the sinusal level.^42^, ^43^, ^44^ Indeed, some studies underscored the potential of a “root-phenotype” in BAV patients to address a peculiar genetic and clinical profile.^45^, ^46^, ^47^ Due to the autoptic nature of our study, we cannot draw any conclusion regarding the potential link between root-phenotype and TAD risk. Interestingly, however, in the only 2 BAV patients in whom previous echocardiography was available, the largest aortic diameter was detected at the tubular level, and at autopsy the case with “complicated” BAV with previous surgery for coarctation showed increased aortic size.

### Histopathologic changes of the aorta in SCD-TAD

To assess whether a characteristic histological substrate predispose BAV patients to dissect (ie, SMCNL, EFF, and MEMA), we first attempted to apply to postmortem specimens of TAD the grading system of degenerative aortic disease which has been proposed for surgical pathology investigation.^21^ Overall, Marfan cases have more severe medial degeneration than any other group, particularly in terms of translamellar MEMA and EFF. Our data are consistent with those by Waters and coworkers^22^ who found that overall patterns of histopathologic changes could separately cluster BAV and nonsyndromic subjects from Marfan and Loeys–Dietz subjects. Marfan syndrome cases significantly had more overall medial degeneration and MEMA than other syndromes. BAV cases tended to cluster with nonsyndromic cases and away from Marfan and Loeys-Dietz subjects. Overall, a wide range of histopathologic changes does exist for each disease entity.

Underlying genetic alterations have been demonstrated only in a small subset of BAV cases and associated aortopathy.^47^^,^^48^ These results support the potential genetic origin of the associated aortic disease in this specific BAV cohort. The presence of severe degenerative changes of the aortic tunica media in some young BAV patients should raise the need to exclude a coexistent genetic alteration. The identification of fibrillin 1 gene pathogenic variants in BAV patients without Marfan syndrome is fully consistent with the present findings.^49^ The complexity of the potential genetic background of BAV^47^, ^48^, ^49^, ^50^ prompts the need for further studies to elucidate genetic background of BAV-related TAD. However, we support the idea that, in the absence of family history of TAD and/or features suggesting a syndromic phenotype, direct referring of each BAV patient with thoracic aortic aneurysms for a clinic-genetic assessment is impractical and unsustainable, due to the prevalence of BAV in the general population and its common association to significant aortic dilatation.^43^

### Study Limitations

There are some limitations to this study. The total number of prospectively collected SCD-TAD cases is low, thus limiting the generalizability of our findings; however, the study is the only population-based study of TAD-SCD in a well-defined geographic area, thus allowing to provide data about the incidence of TAD-SCD in 1,000,000 young inhabitants <40 years old. Not all patients underwent genetic test, as such, some genetic diseases could have been missed and more BAV-TAD cases could have been labeled “complicated BAV.” Since the study began almost 40 years ago, it is conceivable that in some patients with an in vivo diagnosis updated diagnostic procedures, genetic testing and therapeutic advances were not available as well as the awareness of the potential of early surgery. Aortic size was measured at postmortem and not by computed tomography or magnetic resonance. However, a coefficient was applied to all the aortic measurements to correct the possible relative underestimation due to the absence of perfusion.

## Conclusions

The incidence of SCD due to TAD in young individuals is very low. BAV is the most frequent associated condition, accounting for one-third of TAD. As compared with Marfan syndrome, BAV patients are characterized by small aortic diameters and a histopathology pattern of the tunica media rarely characterized by severe EFF and MEMA. Since preceding symptoms are not that rare, a high index of clinical suspicion is needed for timely diagnosis. The high diagnostic yield of genetic screening in young victims of TAD supports its use to identify the molecular basis for the benefit of surviving family members.

Perspectives **COMPETENCY IN PATIENT CARE:** In this 4-decades-long prospective population-based study, the incidence of TAD-related SCD is 0.32 per 1,000,000 young inhabitants, preceding symptoms and chronic TAD are common, and BAV (mostly complex-phenotype) and inherited disorders are the prevalent associated conditions.

**TRANSLATIONAL OUTLOOK:** TAD-related SCD in the young is rare, often associated with genetic disorders and complex BAV. A high index of clinical suspicion is needed for a prompt diagnosis. The high diagnostic yield of genetic test supports its routine use in this population.

## Funding support and author disclosures

Dr De Gaspari is supported by START-SID 2025DCTVSIDPROGETTI-00125 “Molecular pathology of sudden cardiac death”, University of Padua, Padova, Italy. Drs Basso, De Gaspari, and Rizzo are supported by the Registry for cardio-cerebro-vascular pathology, Veneto Region, Venice, Italy. All other authors have reported that they have no relationships relevant to the contents of this paper to disclose.
