# Supplementary material for: A 4-Decade Population-Based Registry of Thoracic Aortic Dissection Causing Sudden Death in the Young
Source: JACC Adv. 2025 Dec 30;5(2):102487. doi: 10.1016/j.jacadv.2025.102487 (PMC12804369; doi:10.1016/j.jacadv.2025.102487)
Supplement: Supplemental_Material [file mmc1.docx]

**Supplemental Material**

**Table S1**. Multiple group comparisons: aortic diameters and histopathologic changes of the ascending aorta tunica media.

|  | **BAV** | **Marfan** | **Hypertension** | **Pregnancy** | **Other** |  |
| --- | --- | --- | --- | --- | --- | --- |
|  | **Median (95% CI)** | | | | | **Kruskal-Wallis test p-value** |
| **Annulus diameter** | 31,3 (95% CI 29,80-34,40) | 34,40 (95% CI 32,50-36,30) | 32,40 (95% CI 26,40-35,90) | 26,40 (95% CI 26,30-26,50) | 24,85 (95% CI 23-35,10) | * (p-value = 0.0441) |
| **Sinusal diameter** | 38,20 (95% CI 34,40-47,80) | 46,20 (95% CI 42,20-50,20) | 36,30 (95% CI 28,60-42,80) | 33,80 (95% CI 33,40-34,20) | 30,90 (95% CI 28,50-37,40) | * (p-value = 0.0323) |
| **Sino-tubular diameter** | 36,30 (95% CI 33,20-45,60) | 45,70 (95% CI 42,40-49,20) | 35,40 (95% CI 27,30-39) | 30,70 (95% CI 30,20-31,20) | 28,05 (95% CI 24,80-42,20) | ns (p-value = 0,1043) |
| **Ascending diameter** | 43,20 (95% CI 34,40-48,20) | 48,30 (95% CI 45,40-51,20) | 31,30 (95% CI 27,50-40) | 30,30 (95% CI 28,20-32,40) | 37 (95% CI 30,20-48,20) | * (p-value = 0,0482) |
| **EFF** | 4 (95% CI 2-6) | 6 (95% CI 6-6) | 2 (95% CI 2-2) | 1 (95% CI 0-2) | 4,5 (95% CI 0-6) | * (p-value = 0,0248) |
| **SMCNL** | 3 (95% CI 2-3) | 2,5 (95% CI 2-3) | 2 (95% CI 2-4) | 2 (95% CI 2-2) | 2,5 (95% CI 2-4) | ns (p-value = 0,6552) |
| **MEMA** | 4 (95% CI 3-6) | 8 (95% CI 6-10) | 2 (95% CI 0-4) | 2 (95% CI 2-2) | 4 (95% CI 2-10) | * (p-value = 0,0420) |

Kruskal-Wallis test was used for multiple group comparisons, as small number of samples for each group and because data do not follow a normal distribution. Data are reported as the median with a 95% confidence interval (CI), providing a robust measure of central tendency and data variability.

Abbreviations: BAV: bicuspid aortic valve; EFF: elastic fiber fragmentation; MEMA: mucoid extracellular matrix accumulation; SMCNL: loss of smooth muscle cell nuclei

**Table S2.** Postmortem genetic testing in formalin fixed paraffin-embedded tissue: gene mutation data in 15 TAD patients*

| **Sex** | **Age** | **Associated condition** | **Gene** | **cDNA** | **AA** | **ACMG rules** | **Score** | **Classification** | **Protein domain** |
| --- | --- | --- | --- | --- | --- | --- | --- | --- | --- |
| M | 35 | BAV | NEG |  |  |  |  |  |  |
| M | 36 | BAV | FBN1 | c.6005C>T | p.(Pro2002Leu) | PM1 (M), PM5 (M), PM2 (S), PP3 (S) | 6 | LP | EGF-like 34 |
| M | 24 | BAV | NEG |  |  |  |  |  |  |
| M | 29 | Familiarity | NOTCH1 | c.4401C>A | p.(Cys1467Ter) | PVS1, PM2 | 9 | LP | LNR 1 (Lin-12/ Notch repeat 1) |
| M | 17 | Idiopathic | TGFBR1 | c.1472G>A | p.(Arg491Gln) | PP5 (VS), PM5 (Strong), PP3 (Strong), PM1 (M), PM2 (S) | 19 | P | Protein kinase |
| M | 38 | Idiopathic | NEG |  |  |  |  |  |  |
| M | 39 | Idiopathic | MYLK | c.4415+1G>C | / | PVS1 (VS), PM2 (S) | 9 | LP | / |
| M | 31 | Marfan | FBN1 | c.5930G>A | p.(Cys1977Tyr) | PM5 (strong), PP3 (strong), PP5 (strong), PM1 (M), PM2 (Supporting) | 15 | P | EGF-like 34 |
| M | 28 | Marfan | FBN1 | c.4011delT | p.(Val1338TyrfsTer75) | PVS1 (VS), PP5 (Strong), PM2 (Supporting) | 13 | P | EGF-like 22 |
| F | 30 | Pregnancy | COL3A1 | c.3005G>A | p.(Gly1002Asp) | PM1, PM2, PP3(S) | 9 | LP | Triple-helical region |
| M | 39 | HPT | NEG |  |  |  |  |  |  |
| F | 40 | HPT | NEG |  |  |  |  |  |  |
| M | 40 | HPT | NEG |  |  |  |  |  |  |
| M | 32 | HPT | NEG |  |  |  |  |  |  |
| M | 33 | Drugs | NEG |  |  |  |  |  |  |

*Abbreviations*: AA: amino-acid; ACMG: American College of Medical Genetics and Genomics; BAV: bicuspid aortic valve; COL3A1: collagen type III alpha 1 chain; EGF-like: Epidermal Growth factor; FBN: fibrillin; HPT: hypertension; LNR1: Lin-12/Notch repeat 1; LP: likely pathogenetic; MYLK: myosin light chain kinase gene; NOTCH1: notch receptor 1; P: pathogenetic; TGFBR1: transforming growth factor beta receptor 1.  * An additional patient (F, 34) had Turner Syndrome, chromosomal abnormality 45X0.
